# The Synergy between Pharmacological Regimens and Dermocosmetics and Its Impact on Adherence in Acne Treatment

**DOI:** 10.1155/2022/3644720

**Published:** 2022-08-09

**Authors:** Elena Araviiskaia, Alison Margaret Layton, Jose Luis López Estebaranz, Falk Ochsendorf, Giuseppe Micali

**Affiliations:** ^1^First Pavlov State Medical University, St Petersburg, Russia; ^2^Harrogate and District NHS Foundation Trust Harrogate, North Yorkshire, UK; ^3^University Hospital Fundación Alcorcón, Madrid, Spain; ^4^Clinic for Dermatology, Venereology and Allergology, University Hospital Frankfurt, Goethe University, Frankfurt, Germany; ^5^University of Catania, Catania, Italy

## Abstract

**Background:**

Acne is the most common inflammatory skin disease in adolescence. It is also prevalent in adults, especially females. The disease has a considerable impact on health-related quality of life. Many studies have reported the negative impact of acne on patients due to skin disfigurement, ineffective treatment, and adverse effects of the treatment. Numerous factors contribute towards nonadherence to therapy. *Summary*. This review discusses the various factors that are related to treatment nonadherence such as ineffective therapy, adverse effects with topical pharmacotherapy such as skin irritation and erythema as well as patient-related factors such as lack of knowledge of disease and a poor patient-physician relationship. Various methods are being adopted to increase adherence to treatments. Increased adherence to acne therapy has been associated with the use of dermocosmetics, such as moisturizers and cleansers. Encouraging the use of dermocosmetics in synergy with pharmacological regimens could support improved treatment adherence resulting in better clinical outcomes for acne patients.

**Conclusion:**

Dermocosmetics as an adjunct to pharmacological regimens has the potential to improve clinical outcomes by increasing treatment adherence in patients with acne.

## 1. Introduction

Acne is a multifactorial skin disorder affecting approximately 9% of the world's population [1]. Though acne is predominant in adolescence, there has been an increase in the incidence of this disease in the adult population, especially females [2, 3]. The systematic analysis of the Global Burden of Disease Study 2010 showed an acne prevalence of 9.81% in females and 8.96% in males [1, 4].

The intensity of acne is moderate to severe in 15–20% of the affected population [5]. Acne is associated with negative impacts on mental health; patients get frustrated, embarrassed, and distressed due to the disease's sequelae [6–11].

The primary goal of the treatment is to address the immediate clinical signs and symptoms and prevent sequelae such as pigmentation and physical and “psychological” scarring. The selection of pharmacotherapy is based on the intensity and duration of acne, evidence of sequelae, characteristics of the patient, and patient's preferences and expectations from the treatment [12].

Nonadherence to the prescribed treatment plan can impact treatment outcomes and lead to financial burden. A large-scale observational study among 3339 acne patients from America, Europe, and Asia showed a poor adherence rate of 50% [13].

Dermocosmetics are being adopted for the management of acne to achieve optimal clinical outcomes, improve adherence, and minimize the side effects that can arise from pharmacotherapy [14–16]. Dermocosmetics can provide additional value to the medical regimens prescribed for acne and can reduce local inflammatory reactions associated with topical medications [17]. Among the vast range of cosmetics available, there is an expanding group of products that have undergone more rigorous clinical testing and have demonstrated efficacy and safety when used to treat specific skin problems [18]. Novel dermocosmetics are being developed and increasingly adopted as an adjunct in the management of acne [18, 19].

The aim of this review is (i) to provide an overview of the synergy between pharmacological regimens and dermocosmetics, (ii) to review the role of dermocosmetics to improve treatment outcomes and increase patient adherence to the treatment, and (iii) to consider how dermocosmetics can target additional pathogenic factors to improve treatment outcomes. In addition, other measures used to enhance treatment adherence are discussed.

## 2. Methodology

This article results from a focus meeting on acne disease burden, treatments, and their shortcomings including lack of adherence to prescribed treatments. The meeting was held in April 2021 and was attended by all the authors to reach consensus on the role of dermocosmetics in the context of acne management, with a specific focus on the synergy between pharmacological regimens and dermocosmetics and its impact on adherence in acne treatment.

## 3. Pharmacological and Dermocosmetics in Management of Acne

### 3.1. Pharmacological Regimens in Acne

The management of acne using pharmacological regimens depends on numerous factors, including disease severity, the site affected, duration of disease, presence of sequelae, age, and gender of the patient, as well as treatment preferences. Topical and oral retinoids, antibiotics (clindamycin, erythromycin, and tetracycline), azelaic acid, and benzoyl peroxide (BPO) represent some of the available therapies for acne treatment [20–22].

#### 3.1.1. Topical Monotherapies in Acne

Topical monotherapies for acne include retinoids, azelaic acid, and antimicrobials. The European evidence-based (S3) guidelines recommend topical retinoids for the treatment of comedonal acne, while azelaic acid and BPO can also be considered [11]. A meta-analysis of five randomized trials demonstrated equivalent efficacy of adapalene 0.1% compared to tretinoin 0.025% gel for reducing lesion count but better local tolerability of adapalene at all study visits [23]. A recent study showed that BPO monotherapy or add-on therapy resulted in better efficacy than placebo or no treatment [24]. Due to the serious concerns regarding the risk of developing antibiotic resistance, topical monotherapy with antibiotics such as erythromycin and clindamycin is not recommended [11]. Combination therapy is recommended to effectively control acne by targeting multiple underlying pathological factors [11].

#### 3.1.2. Topical Combination Pharmacological Regimens in Acne

Topical fixed combination therapies are advocated as first-line treatment by the European evidence-based (S3) guideline for the treatment of acne [11, 20]. These products have been shown to achieve rapid and superior efficacy when compared to individual monotherapies and result in improved adherence [11, 25].

BPO is associated with concentration-dependent adverse events such as irritation and dryness that lead to poor patient adherence [24, 26]. However, various studies on moderate to severe acne have demonstrated that the combination of BPO 2.5% gel and clindamycin 1.2% gel as a fixed dose has better efficacy and tolerability compared to BPO 2.5% gel alone [26, 27]. Both topical retinoids and BPO have an anti-inflammatory effect. Topical retinoids inhibit microcomedone formation, normalize keratinocytes desquamation, and topical antibiotics inhibit the growth and activity of *C. acnes* [12]. Combinations of retinoids and BPO are associated with increased sensitivity of the skin to sunlight, skin irritation, prolonged erythema, and peeling [28–30]. Patients using retinoids + BPO are advised to minimize sun exposure and apply sunscreen for daily protection when exposed to ultraviolet (UV) radiation [25, 30, 31]. In a trial with 480 patients subjected to 5% benzoyl peroxide + 1% clindamycin, 5% benzoyl peroxide, 1% clindamycin, or vehicle treatment, 5% benzoyl peroxide + 1% clindamycin showed better reductions in the number of inflammatory and total lesions. However, the AEs were similar to that of the BPO arm [32]. In another pooled analysis of 3 clinical trials, fixed-dose adapalene 0.1%-benzoyl peroxide (BPO) 2.5% was efficacious for the treatment of patients with a high number of acne lesions [33].

Consensus guidelines from the Global Alliance and European Dermatology Forum recommend fixed combination topicals including a retinoid and an antimicrobial (which may be an antibiotic) as first-line therapy for mild to moderate forms of acne and then an oral antibiotic for more moderate to severe disease [11, 31]. The combination of these active ingredients enables three out of the four major pathogenic factors of acne to be targeted, namely abnormal keratinization, bacterial hypercolonisation, and inflammation.

The use of topical retinoids such as tretinoin, tazarotene, adapalene, and trifarotene may lead to acne flares during the first few weeks of treatment [34]. The inflammatory symptoms may include skin dryness, erythema, and pain [35]. A meta-analysis of 54 clinical trials, evaluating both efficacy and safety/tolerability of topical retinoids, has shown that almost 62% of the patients experienced local skin irritation while using tretinoin at 0.05% [36]. The incidence of acne flare, one of the most important causes of treatment discontinuation among patients using topical retinoids, was reported to be lowest with Clin-RA when compared to monotherapy and vehicle. Also, the combination showed a better tolerability profile in terms of cutaneous side effects [37]. Compared to monotherapy with topical retinoids, a combination therapy of antibiotic and retinoid, or antibiotic and BPO, is associated with fewer side effects and may have better treatment adherence.

Fixed topical combination therapy with clindamycin 1% + tretinoin 0.025% (Clin-RA) showed better efficacy and safety compared to the individual components in mild to moderate cases of acne vulgaris [27, 38–41]. The latest NICE (National Institute for Clinical Excellence) guidelines recommend a fixed combination of (a) topical adapalene with topical benzoyl peroxide, (b) topical tretinoin with topical clindamycin, or (c) topical benzoyl peroxide with topical clindamycin for mild to moderate acne [42]. A pooled analysis of phase III studies in which 4550 patients were randomized to receive clindamycin (1428), tretinoin (846), Clin-RA (1853), or vehicle (423) showed Clin-RA to be an effective and safe acne treatment. Retinoids normalize keratinization and facilitate the penetration of the antibiotic into the sebaceous follicle. Due to this mechanism of action, the combination (retinoid/antibiotic) did not show antibiotic resistance over a “12” week trial period [22, 31, 43, 44].

Topical retinoids and BPO can cause increased transepidermal water loss (TEWL), impaired permeability of stratum corneum (SC), and increased sensitivity of the skin to UV radiations [17]. In addition, BPO can cause a decrease in stratum corneum vitamin E [45]. Possible adverse events of topical retinoid therapy include erythema, dryness, pruritus, stinging sensation, and photosensitivity, which can lead to poor adherence. To minimize skin irritation, dermocosmetic agents have been recommended and shown to improve adherence to these treatments [46, 47].

## 4. Dermocosmetics in Acne

Dermocosmetics are used as adjuncts to pharmacological therapies for acne and can be formulated to target the main pathogenic pathways in acne. Dermocosmetics may minimize the side effects of acne medications and provide a synergistic effect by targeting additional pathogenic factors causing acne and/or improving the efficacy of other treatments [48]. Effective management of acne should comprise two phases: an initial treatment phase, previously described, where medication is prescribed to reduce the extent and severity of acne lesions, and a maintenance treatment phase, aimed at preventing relapses. Particular ingredients used in some dermocosmetics are known to target the different acne pathways (Table 1) and,as such, can be used as a maintenance treatment option. In this way, it would be possible to maintain the level of improvement and prevent the appearance of new lesions in addition to targeting specific aspects of acne pathogenesis [18, 48]. Topical products are also used to camouflage the disfigured skin and improve its appearance [47, 49].

### 4.1. Dermocosmetics Targeting Excess Sebum Production

Excess sebum production is one of the most important pathogenic pathways in acne. Dermocosmetics targeting excess sebum production are sebo absorbents (i.e., absorbing excess sebum) that cause direct seboregulation or affect sebum composition. Few topical products such as nicotinamide, fullerene, epigallocatechin-3-gallate, and triethyl citrate + ethyl linoleate + salicylic acid reduce the skin's surface sebum level [18, 48]. Acrylate copolymer technology is used for the absorption of excess sebum from the skin [50]. L-carnitine also effectively reduces excess sebum production. In an in vitro study in human SZ95 sebocytes, L-carnitine effectively reduced intracellular lipid content. In addition to sebum-controlling effects, these agents also have antipruritic, antimicrobial, vasoactive, photoprotective, skin-lightening, antioxidant, and anti-inflammatory activities [49]. A 9-week randomized, double-blind study in 30 patients showed a reduction in the lesion count, skin surface sebum levels, and *C. acnes* colonization after application of an emulsion containing licochalcone A, L-carnitine, and 1,2-decanediol [51]. From some preliminary data, 1, 2-decanediol also seems to have an activity in reducing skin sebum content [15, 52].

### 4.2. Dermocosmectics Targeting Abnormal Keratinization

To stimulate the new epidermal growth, exfoliating agents such as alpha hydroxy acids (AHA) and salicylic acids (SA) are used for superficial peeling in acne patients. A randomized controlled study showed a significant decrease (*p* < 0.001) in the size and number of microcomedones in women with acne [53].

### 4.3. Dermocosmetics Inhibiting *C.acnes* Colonization and Reducing Inflammation

*C.acnes* colonization leads to the release of inflammatory mediators, resulting in inflammation and scarring. Nicotinamide and *Salix alba* in combination with 1,2 decanediol and soy isoflavones are some of the ingredients in dermocosmetics that inhibit *C.acnes* colonization and/or have anti-inflammatory effects [54]. Studies investigating moisturizers with antiacne pharmacotherapy reported significant improvement in adverse effects of pharmacotherapy (such as erythema, inflammation, and skin irritation) and increased treatment compliance [47, 55–60]. A study (*in vivo* and *in vitro)* was conducted to evaluate the effect of nicotinamide (NAM) on biofilms of *C. acnes*. NAM decreased the formation of biofilms when used with suboptimal dosing of tetracycline [61].

The effects of various dermocosmetics used in the form of creams, gels, and lotions are described in Table 1. A total of 596 dermatologists of Brazil were interviewed to understand the prescribing practices for acne patients. The majority of skin specialists prescribed more than one type of agent for acne pharmacotherapy depending upon the severity of the disease. Antibiotics and isotretinoin were considered by most dermatologists. Dermocosmetics were given as an adjunct treatment; however, with an increase in the severity of acne, the use of dermocosmetics was decreased [62]. Large clinical studies on the use of dermocosmetics to evaluate their efficacy and safety in comparison to placebo are still lacking.

### 4.4. Safety of Dermocosmetics

The development and manufacturing of dermocosmetics follow stringent regulatory standards to ensure patient safety [19]. Multiple ingredients such as mineral oils, waxes, glycerine, and octocrylene are used in this kind of product. For example, mineral oils used in these products are of high-grade purity, conforming to EC/1223/2009 regulations of the European pharmacopoeia and the EU cosmetics regulation. These ingredients have an excellent safety profile and are not systemically bioavailable to the body [63]. All safety assessments (prelaunch) and postmarketing surveillance (postlaunch) for a given cosmetic formulation are carried out by the manufacturers, which is a similar process to medical products and devices. Postmarketing surveillance of cosmetic products entails the monitoring by companies and competent authorities of the safety of products on the market [19].

### 4.5. Pharmacoeconomics of Dermocosmetics

Sales in the United States alone for cosmeceutical products are expected to increase by 7.4% per year. An average consumer in the United States uses at least 25 products containing hundreds of ingredients on their skin daily [64]. Considering that dermocosmetics are used as adjuncts to pharmacological and light therapies for acne due to their specific formulation targeting the main pathogenic pathways of the disease, their use could be even greater in these types of patients [48]. The use of dermocosmetics must be placed in a more general framework in which it has been estimated that more than 11 million prescriptions per year are written for the treatment of acne and that acne therefore imposes a significant burden on healthcare systems and economies. In 2004, the total annual cost relating to acne in the USA was evaluated at $3.1 billion. Considering their role in the management of the disease, dermocosmetics can be fully included in the so-called intangible costs, reflecting the patient's willingness to pay for the alleviation of symptoms associated with their acne [65]. Bickers et al. [66] found that the intangible cost of acne was $12 billion in the USA in 2004. Compared with other leading skin diseases, patients with acne show a much higher willingness to pay than those with atopic dermatitis, herpes simplex, or psoriasis [65].

## 5. Synergistic Effects of Dermocosmetics and Pharmacotherapy

Dermocosmetic agents are prescribed based on the disease severity and patient characteristics. Controlled studies evaluating dermocosmetics as an adjuvant to pharmacological treatment for better adherence and improved clinical outcomes in acne patients are limited. In a double-blind, placebo-controlled, randomized, multicentre trial, 140 patients with moderate acne received either adapalene and nicotinamide + antibacterial adhesive agents + zinc-pyrrolidone carboxylic acid (Arm A) or adapalene and a placebo cream (Arm B) twice daily for six weeks. There was a significant reduction (*p* < 0.05) in noninflammatory lesions in arm A [67]. In another double-blind study, both 5% nicotinamide and 2% clindamycin reduced the acne severity index (ASI) significantly (*p* < 0.0001) as compared to baseline after treatment for 8 weeks [68].

A new topical cream (Acne RA-1,2) containing active ingredients such as 1,2-decanediol, *Salix alba*, UV filters, and vitamins B3, E, and C has demonstrated efficacy and safety in a real-life study in 40 patients under the acne pharmacological regimen [15]. The study showed a reduction of 38% in the Investigator's Global Assessment (IGA) score, a 29% reduction in TEWL, and a 17% reduction in sebum production as compared to baseline. In addition, it showed a significant improvement in tolerability scores for pruritus, erythema, and dryness, which represent the main local side effects caused by pharmacological acne treatments [15]. A key result of this study was the demonstration that the synergist use of Acne RA-1,2 with pharmacological therapy led to 100% of patients that were adherent to their pharmacological acne treatment [15].

The use of dermocosmetics as part of an acne regimen could result in better clinical outcomes and adherence to acne therapy. Various dermocosmetics such as cleansers (containing benzoyl peroxide or azelaic/salicylic-acid/triclosan), sebum-controlling agents (containing nicotinamide or zinc acetate), and antimicrobial or anti-inflammatory cosmetics should be strongly recommended in all acne patients in addition to the pharmacological regimen [69]. The product has beneficial effects on acne (anti-inflammatory, antimicrobial, sebum reduction, and skin barrier protection) [15, 16]. The ACTUO observational prospective study conducted on 643 patients found that the use of dermocosmetics (cleansers, moisturizers, emollients, and lenitive specific topical products) increased treatment adherence and resulted in better clinical outcomes [14]. Hence, dermocosmetics could provide a synergistic effect by targeting additional pathogenic factors and/or improving the efficacy of treatments prescribed for acne by a clinician. When patients are left free to choose the product of their liking, it frequently leads to acne worsening likely from the use of inadequate comedogenic products or to the onset of xerosis, irritant/allergic contact dermatitis, or photodermatitis resulting from overtreatment [69]. Thus, the use of dermocosmetics as advised by the prescribing clinician is an important part of acne management and could improve patient adherence, leading to better clinical outcomes [18].

## 6. Treatment Adherence

### 6.1. Treatment Adherence: Problem Statement and Associated Factors

Acne patients' adherence to treatment is poor [13]. An international study assessed the risk of poor adherence to acne treatments in 3339 patients, using a validated questionnaire. The results confirmed a poor adherence rate of 50%; this varied by region, with significantly worse adherence in Europe versus Asia and America (poor adherence rates of 58%, 48%, and 43%, respectively, *p* < 0.0001) [13]. In addition, among patients taking a combination of both systemic and topical therapy, 60% (*n* = 944) of patients had poor adherence to at least one treatment, and among patients treated with topical therapy only, poor adherence occurred in 40% (*n* = 356) of cases [13].

**Figure 1:**
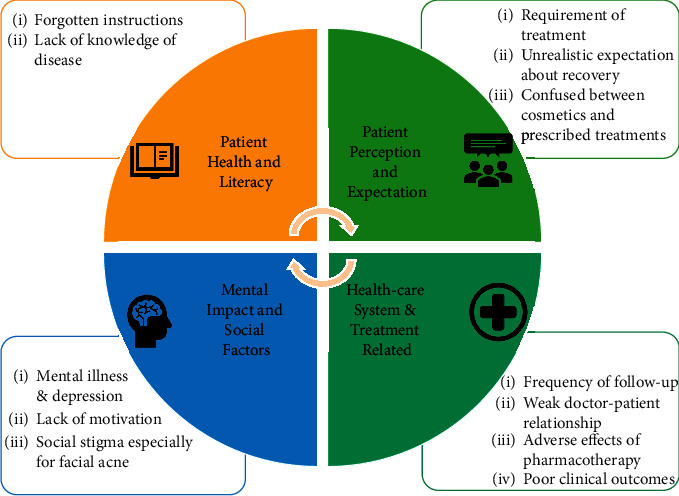
Factors associated with poor adherence.

Poor treatment adherence is associated with two main factors: lack of knowledge about the acne treatment and adverse effects of the treatment [46]. It has been reported that a younger age group does not adhere to treatment as well [70–73]. Literature has shown that generally the number of treatments prescribed plays a role in the level of adherence, with higher numbers of treatments leading to poorer adherence [74]. The treatments fitting to the patient's lifestyle and less complex regimens lead to improved adherence. Other factors that lead to better adherence include topical therapies with acceptable vehicles, treatments that achieve rapid clinical improvement and once-daily regimens [13, 75]. Fixed dose combination therapies are more convenient for patients to apply than applying two or more separate formulations [31, 76]. Hence, adherence may be improved by decreasing the complexity of the regimen, minimizing side effects of acne medication, and improving patient satisfaction with topical formulations that contain multiple active ingredients [77].  Figure 1 summarizes the factors associated with nonadherence.

### 6.2. Measures towards Improvement in Adherence

Patients' health literacy is vital in ensuring medication adherence. However, other supportive interventions play a critical role. Some of the interventions include electronic messages and phone calls, virtual engagement with a wider team, audio-visual/internet-based interventions, and patient support programs, which are highly effective in improving treatment adherence [78, 79]. Another important factor that influences adherence to treatment is the doctor-patient relationship. One of the most important reasons for lack of adherence relates to failure on the clinician's part to appreciate the patient's perspective, which significantly influences the “doctor-–patient relationship.” The physician should be approachable and motivational [80]. The physician should be able to understand what the barriers to patients' adherence to prescribed acne medications are. Understanding these hurdles may help dermatologists in creating effective solutions for their patients [81].

**Figure 2:**
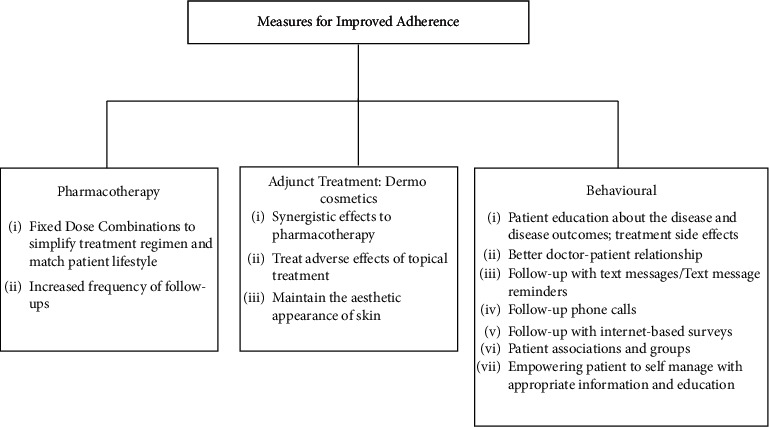
Methods to improve adherence.

From the literature, we know that during the interview with the patient, it is useful to investigate and address the most frequent causes of primary and secondary nonadherence such as little knowledge about the severity of acne, influence from the media or other physicians, fear of adverse reactions, or confusion about how to use treatment [82]. Other factors to increase adherence include frequent and convenient clinic visits [13, 66] and simplified treatment regimens [66]. Recent restrictions due to the pandemic prevented face-to-face consultation with the clinician for nonemergency medical conditions. Teledermatology not only helps the patients to consult the clinician easily but also enables the clinician to remotely monitor the patients regularly, ensuring better adherence [83]. The measures used to obtain an improved adherence to treatment relating to pharmacotherapy, adjunct treatments, and behavioural aspects are summarized in Figure 2.

## 7. Conclusions

Dermocosmetics as an adjunct to pharmacological regimens improve clinical outcomes, increasing patient adherence. There are different types of dermocosmetic preparations, some of which can provide additional benefits, and others that can worsen the acne condition.

Generally, the safety level of dermocosmetics specifically developed for the management of acne is good, thanks to the high quality of the ingredients that compose them, and the close surveillance carried out prelaunch and postlaunch.

The strong impact of the disease, not only from a morphological but also a psychological point of view, pushes patients to face overall high costs for improving their quality of life. Dermocosmetics fall within the so-called intangible costs but have the potential to improve the condition of these patients in association with correct medical treatment. Thus, the use of dermocosmetics should be advised by the prescribing clinician as an important part of acne management. There is still an unmet need to better understand a patient's expectations and educate people regarding acne to achieve better treatment outcomes. Therefore, adopting dermocosmetics as part of routine acne care provides a positive step towards improved patient outcomes.

## Figures and Tables

**Table 1:** Dermocosmetic Ingredients targeting pathogenic pathways of Acne [18].

Agent	Anti-inflammatory	Sebum control	Antimicrobial activity	Keratolytic	Antioxidants
Salix alba	√				
Decanediol	√		√		
Nicotinamide	√	√			
Epidermal growth factor	√				
Tea tree oil			√		
Epigallocatechin-3-gallate		√			√
Fullerene		√			√
Alpha-hydroxy acid				√	
Salicylic acid				√	
Retinol derivatives				√	
Glycolic acid				√	
Lipohydroxy acid				√	
Linoleic acid				√	
Vitamin C					√
Vitamin E					√

Table modified by Araviiskaia E, Dreno B. The role of topical dermocosmetics in acne vulgaris. Journal Eur Acad Dermatol Venereol. 2016; 30(6):926–35 with the elimination of soy isoflavones, according to the information in European Commission (2022) SCCS - Opinions 2016–2021 - preliminary opinions open for comments available at: https://ec.europa.eu/health/scientific-committees/scientific-committee-consumer-safety-sccs/sccs-opinions-2016-2021_en (Accessed: Jan. 21, 2022).

## Data Availability

The data used to support the findings of this study are included within the article.
